# Hierarchical classification of snowmelt episodes in the Pyrenees using seismic data

**DOI:** 10.1371/journal.pone.0223644

**Published:** 2019-10-10

**Authors:** Jordi Díaz, Pilar Sánchez-Pastor, Mario Ruiz

**Affiliations:** Institute of Earth Sciences Jaume Almera, Consejo Superior de Investigaciones Científicas (ICTJA-CSIC), Barcelona, Spain; University Hospital Eriangen at Friedrich-Alexander-University Erlangen-Numberg, GERMANY

## Abstract

In recent years the analysis of the variations of seismic background signal recorded in temporal deployments of seismic stations near river channels has proved to be a useful tool to monitor river flow, even for modest discharges. The objective of this work is to apply seismic methods to the characterization of the snowmelt process in the Pyrenees, by developing an innovative approach based on the hierarchical classification of the daily spectrograms. The CANF seismic broad-band station, part of the Geodyn facility in the Laboratorio Subterráneo de Canfranc (LSC), is located in an underground tunnel in the Central Pyrenees, at about 400 m of the Aragón River channel, hence providing an excellent opportunity to explore the possibilities of the seismic monitoring of hydrological events at long term scale. We focus here on the identification and analysis of seismic signals generated by variations in river discharge due to snow melting during a period of six years (2011–2016). During snowmelt episodes, the temporal variations of the discharge at the drainage river result in seismic signals with specific characteristics allowing their discrimination from other sources of background vibrations. We have developed a methodology that use seismic data to monitor the time occurrence and properties of the thawing stages. The proposed method is based on the use of hierarchical clustering techniques to classify the daily seismic spectra according to their similarity. This allows us to discriminate up to four different types of episodes, evidencing changes in the duration and intensity of the melting process which in turn depends on variations in the meteorological and hydrological conditions. The analysis of six years of continuous seismic data from this innovative procedure shows that seismic data can be used to monitor snowmelt on long-term time scale and hence contribute to climate change studies.

## Introduction

Since the beginning of the seismic instrumental era it has been known that seismometers are able to record natural and human-made phenomena distinct from earthquakes, including earth tides, oceanic waves surf, atmospheric disturbances or human activity [1,2]. However, only in the last decade the analysis of the ground movement recorded in the time intervals without earthquake-generated wave arrivals, commonly referred to as “ambient seismic noise”, has become an important topic, as these vibrations are now widely used in tomographic studies of the subsurface and to provide new insights into a wide range of physical phenomena occurring in the atmosphere, the cryosphere and the hydrosphere, an approach already known as “Environmental seismology” [3]. Ambient seismic noise has been used to study climate variability [4] or to track hurricanes and other large atmospheric perturbations [5]. Cryospheric phenomena investigated using seismic noise include estimations of Antarctic sea ice variability [6] or identification of “icequakes” (glacial earthquakes) associated with the detachment of icebergs [7]. Regarding the hydrosphere, seismic noise has been used, among other objectives, to monitor the oceanic wave height at global-scale [8], to document changes in the depth of the water table [9] or to investigate the different processes occurring at rivers [10].

The analysis of seismic signals generated by rivers is typically done in two different settings. In one hand, steep mountain torrents and Alpine-style streams with low background discharge affecting sensitive civil infrastructures have been investigated seismically to detect and characterize debris flows (rapid landslides of water and poorly sorted solid material) and debris floods (very rapid surging flows of sediment-laden water in steep channels) [11–13]. On the other hand, rivers with large discharge (natural or anthropogenic) have been studied to understand the source processes leading to the seismic noise. As examples, seismic records have allowed to document clear seasonal variations related to the monsoon in Himalayan rivers [14], the passage of great storms (typhoons), with associated discharges ranging from 100 to 4000 m^3^/s in Taiwan [15] and controlled flood experiment in the Grand Canyon with peak discharges reaching 1300 m^3^/s [16].

**Fig 1:**
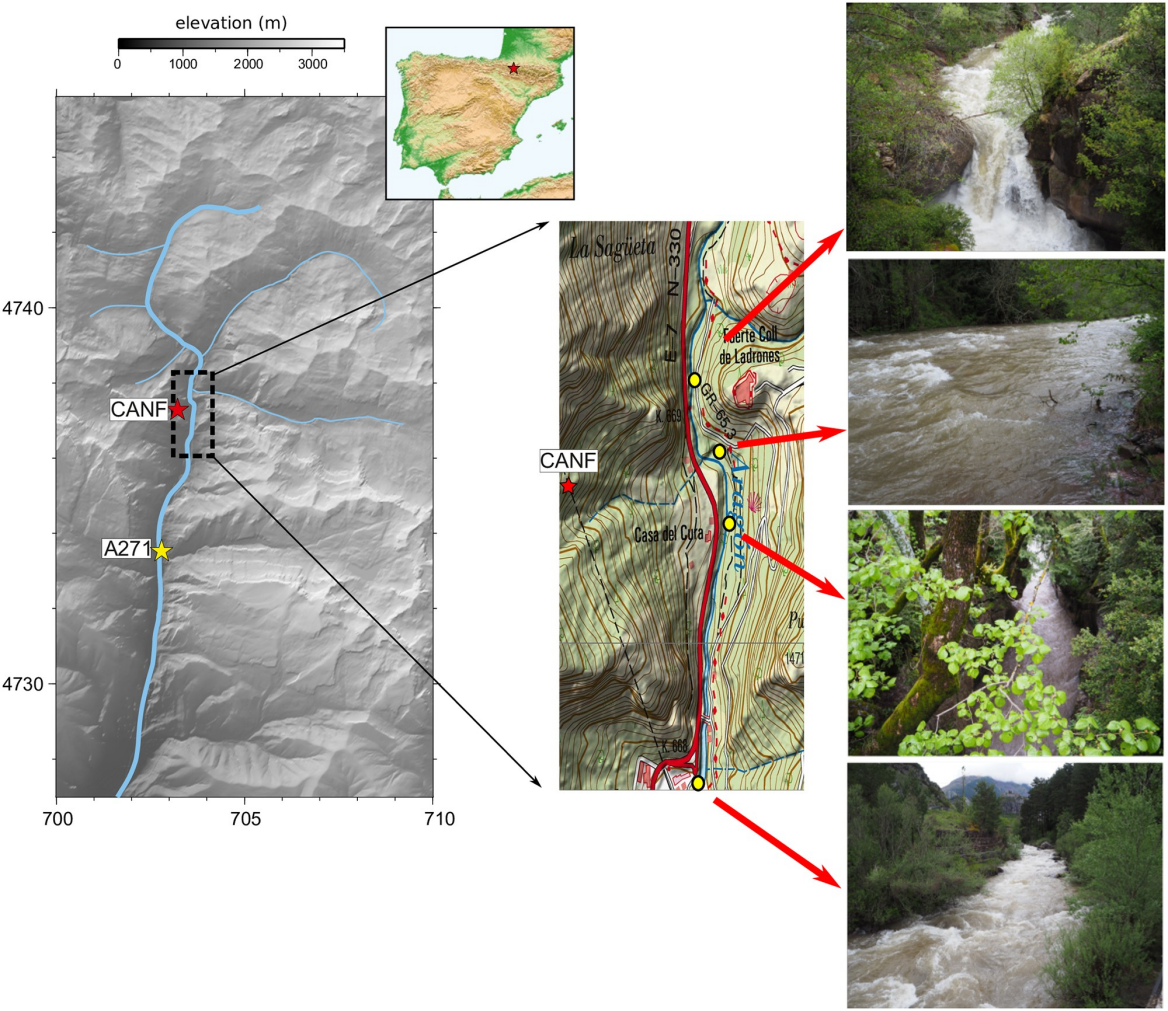
Situation map. Left panel: Map of the upper section of the Aragón River. The locations of the CANF seismic station and the A271 gauge station are shown using red and yellow stars. Digital elevation model is provided by the Instituto Geográfico Nacional with a 25 m resolution (http://www.ign.es/ign/layoutIn/modeloDigitalTerreno.do). Coordinates are labeled in km (Universal Mercator projection). Right panel: Blow up map and pictures showing the morphology of the river channel in the vicinity of the recording site. Yellow dots show the location of each picture, all of them taken upstream.

This seismic monitoring of rivers is usually carried on using temporal deployments of seismic stations, extending from few days to some months. The permanent CANF seismic broad-band station (https://doi.org/10.7914/SN/LC), located within an old railway tunnel in the Central Pyrenees, offers an excellent opportunity to extend this view, since it provides a long-term monitoring of the Aragón River, an example of Alpine-style mountain river. This station is part of the Geodyn facility at the Laboratorio Subterráneo de Canfranc (LSC, https://lsc-canfranc.es/en) and is located at about 400 m of the Aragón River channel, one of the left-hand tributaries of the Ebro. The Aragón River rises at 2050 m of elevation, 7 km North of our observation point and gives waters to the Ebro River 195 km away from its origin. The upstream drainage area is around 60 km^2^ and the river channel has an average slope of 5% in the vicinity of the recording site. The discharge at this location is small, with a mean monthly value of 2.65 m^3^/s during the investigated period (http://www.saihebro.com/saihebro/index.php?url=/datos/ficha/estacion:A271). However, severe storms result in discharge peaks reaching 100 m^3^/s that can generate floods with important economic impact on the valley. Along the one kilometer long sector extending north and southward of the minimum distance point, the channel is steep, with a mean slope of 4–8% and small cascading reaches. The channel close to the recording site alternates between sectors deeply entrenched in Paleozoic rocks, where its width is less than 2m, and open sectors where its width reaches 8–10 m (Fig 1).

A previous contribution has documented that the origin of the seismic energy recorded at CANF in the 2–8 Hz frequency range is directly related to the discharge variations in the Aragón River [17]. In this work, we will focus on the seismic signals recorded during snowmelt stages, showing that the number of snowmelt episodes, their duration, the beginning and end of snowmelt season and the different types of episodes according to the intensity of melt can be estimated from the characteristics of the associated seismic records using an innovative methodology based on the use of hierarchical clustering techniques. The availability of data covering 6 snow seasons allows us to compare their variations over a long term scale, hence providing an interesting database for further hydrological studies.

## Data acquisition and processing

The seismic dataset investigated here covers the period 2011–2016. Earth vibration is recorded continuously at 100 samples per second in the three components of a Titan accelerometer and a Trillium 240s broad-band seismometer. This data is uploaded in near real time to a data server and can be accessed openly using the European Integrated Data Archive (EIDA) nodes (i.e. http://orfeus-eu.org/webdc3/), integrated in the EPOS European Plate Observation System. In the following we will focus on the analysis of the records of the vertical component of the broad-band seismometer, although the horizontal components have also been inspected to provide additional information on the backazimuth of the incoming signals. As a first step, the instrumental response is removed from the raw data to correct for the sensor frequency-depending sensibility and the digitizer scaling factor, and the signal is referred to ground acceleration expressed in nm/s^2^.

Although the discharge increases in the Aragón River generated by the snowmelt process results in amplitude variation in the seismic records, those changes are rather subtle and sometimes difficult to identify, and are better identified in the corresponding spectrograms. Spectrograms provide richer information than the time series, as the signal is decomposed to get the time evolution of its frequency content. A color palette, expressed in dB and relative to a reference value of 1 (m^2^/s^4^)/Hz, is used to show the energy distribution, with yellow and reddish colors representing maximum energies.

**Fig 2:**
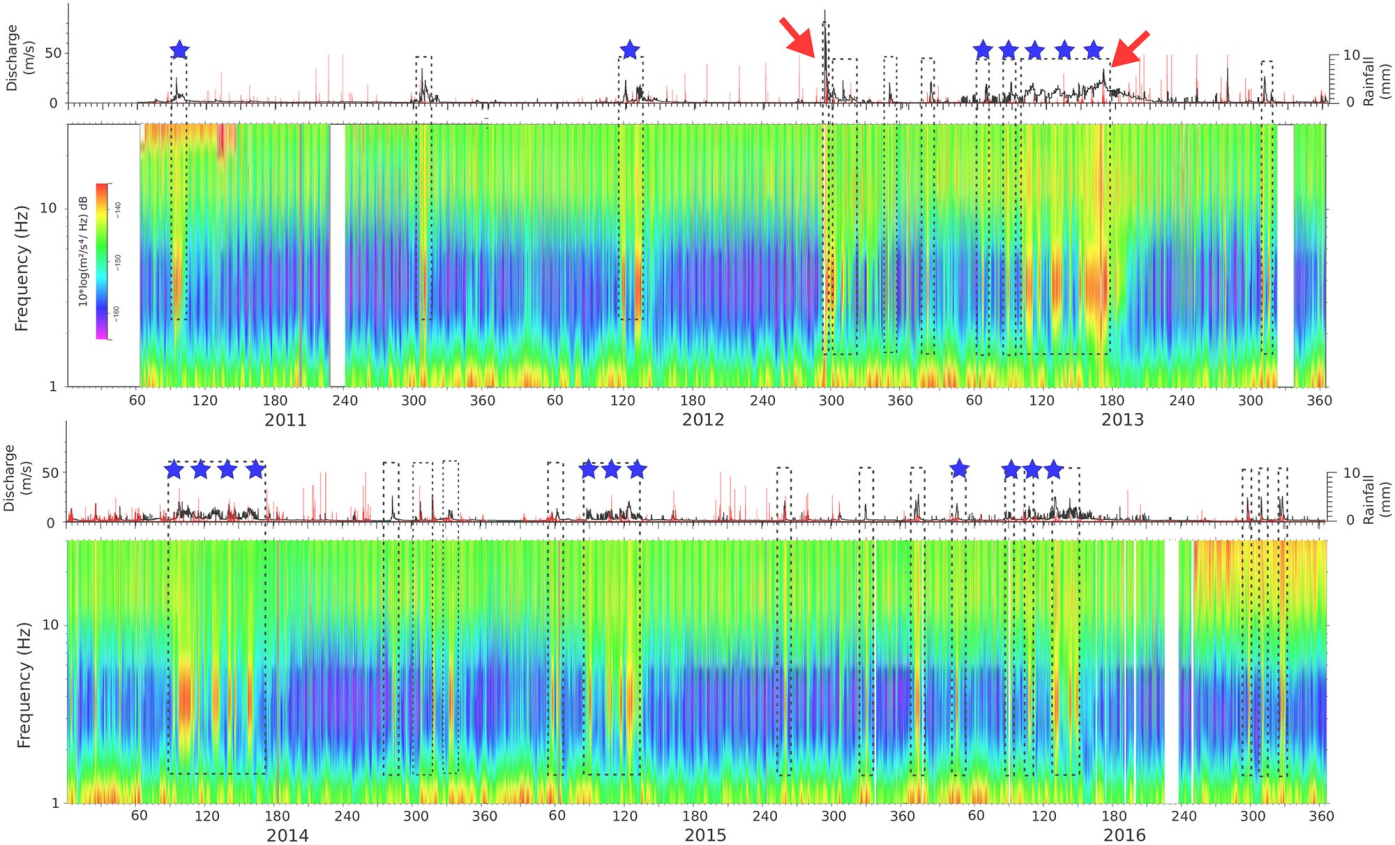
Long-term comparison between discharge, rainfall and seismic energy. Upper panels: Rainfall (red line, mm in 10–15 minutes intervals) and river discharge measurements at the A271 gauge station. Arrows show the main rainfall episodes, while blue stars show the events interpreted as resulting from snowmelt. Lower panels: Power Density Spectra of the vertical component of the seismic data, with reddish colors representing high energy levels. Black dashed boxes show the main hydrologic events and their correlation with intervals of high levels of seismic energy in the 2–10 Hz band. Time scale is in days, with years labeled in the lower axis.

Fig 2 shows the spectrogram of the vertical component of the seismic dataset, calculated using 1 hour long windows and encompassing almost six years of continuously acquired data. This data is compared with the rainfall and river discharge measurements recorded by the A271 gauge station, managed by the Ebro River Basin Authority (Confederación Hidrológica del Ebro, CHE) and located at about 5 km downstream of the seismic station. This gauge station is located downstream of a small pond associated with hydroelectrical production, that can perturb the details of moderate discharge variations. It is easy to identify in the spectrogram shown in Fig 2 the presence of a limited number of episodes of increased energy (reddish colors) in the 2–10 Hz band, mostly occurring in spring and fall and clearly correlated with increases in the river discharge. During late winter and spring, several high-energy episodes can be identified, often lasting several days. Most of those periods correlate with river discharge increases but not with rainfall, and are hence related to snowmelt in the upper Aragón River catchment [17]. Additional support for this interpretation comes from the available accumulated snow estimations (http://www.magrama.gob.es/ca/agua/temas/evaluacion-de-los-recursos-hidricos/ERHIN/default.aspx), that show steep reductions during the same time intervals.

## Seismic identification of snowmelt episodes

Diaz et al. (2014) have shown an example of seismic recording of a snowmelt episode at CANF station. In the published case, corresponding to the 2011 season, the seismic acceleration in the 2–8 Hz frequency band showed a clear 24-hours periodicity and a V-shaped variation of the dominant frequency. As discussed later, the inspection of the snowmelt episodes during the six seasons available so far has proven that these features are common to snowmelt related seismic events and can be used to discriminate between discharge increases related to snowmelt episodes and those due to rainfall based only in the inspection of the seismic data.

**Fig 3:**
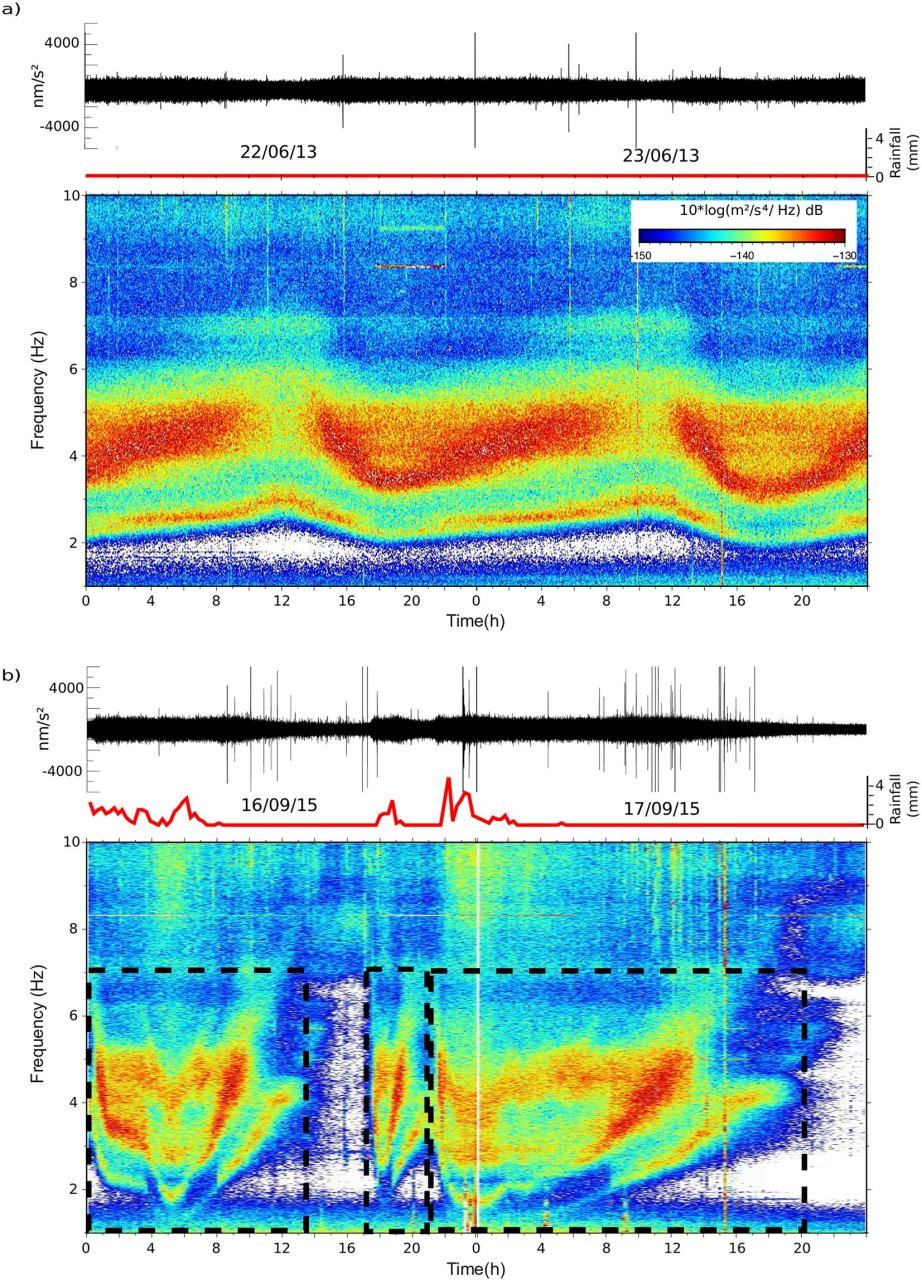
Seismic signatures of rainfall and snowmelt events. Upper panels show the vertical seismic acceleration filtered between 2 and 8 Hz and the rainfall (measured in 15 minutes interval) as recorded at the A271 gauge station (red line, data provided by CHE). Lower panels show the corresponding spectrograms between 1 and 10 Hz. a) Characteristic pattern of a snowmelt event b) Example of rainfall episode including three bursts of precipitation (dashed boxes).

Fig 3 shows a comparison between representative rainfall and snowmelt episodes, the latter being identified by discharge increases without associated rainfall. Although the amplitude of the seismic signal is similar, its time distribution is clearly different, as shown clearly in the spectrograms. During snowmelt episodes (Fig 3a) the seismic signal starts in the afternoon and last for about 22 hours. The dominant frequency decreases steeply at the beginning of the episode and then increases again smoothly. This time evolution is in close agreement with the classical observations using hydrographs (i.e. [18]); the melting process starts with sunlight and needs some time to percolate the snowpack, reach the river and hence increase the discharge. The details on this cycle depend on different factors, including the geometry of the catchment, the total amount of the snowpack and the temperature and humidity. The fall 2015 rainfall episode in Fig 3b shows a different pattern, with up to 3 bursts of signal, lasting roughly 12, 4 and 20 hours. The variations in the dominant frequency are in this case more irregular, as they are related to the changes in discharge following the rainfall intensity variations. The first burst shows a rather symmetric frequency variation pattern, reaching the lower frequencies near the middle of the episode. The second burst shows an abrupt decrease in the dominant frequency in its first section. The last and longer burst reaches the minimum frequency in the first part of the episode and shows a long coda with progressively increasing dominant frequencies.

**Fig 4:**
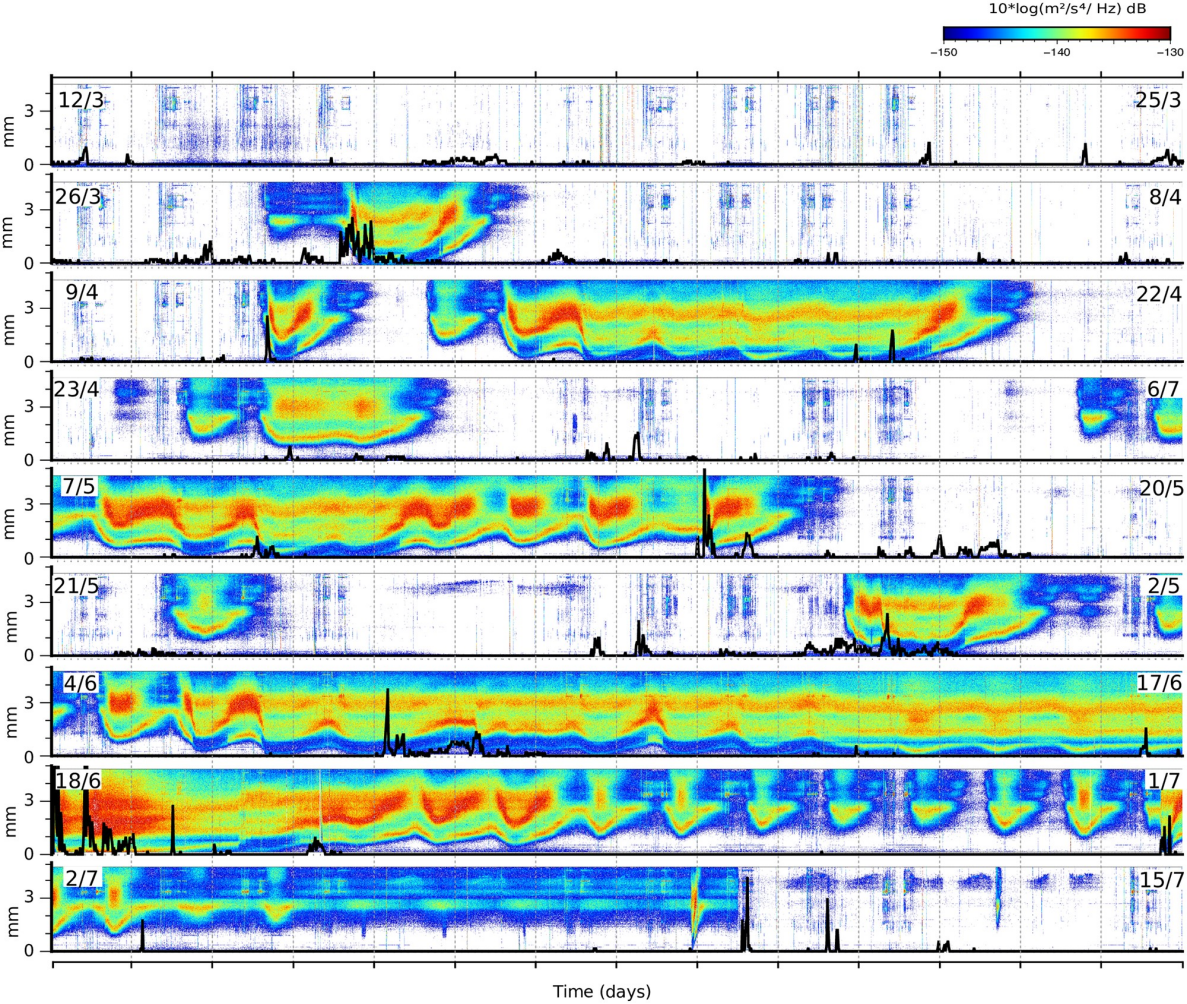
Daily spectrograms during the 2013 snowmelt season (12/3–18/7; Julian days 71–196). Frequency range: 1.5–6.5 Hz. Color scale represents the power spectral density (PSD) expressed in dB relative to 1 (m^2^/s^4^)/Hz, with the reddish colors corresponding to large energy values. Each row shows 14 days, with dates indicated by labels. Black lines show the rainfall accumulated in 15 minutes as measured at the gauge station.

In order to explore the dataset in detail, we have calculated the daily spectrograms for the available time range. Data is gathered in 24 hours long files and, for each file, the spectrogram is calculated using the classical Welch’s power spectral density estimator, with 300 s long time windows and a 50% of overlap. The resulting spectrograms have a time resolution of few minutes, largely enough to discuss the time variations of interest in this study. Fig 4 shows the spectrograms for the period 12^th^ March–15^h^ July of year 2013 (Julian days 80–120) and the rainfall measurement at the CHE gauge station, clearly showing that there is no correlation between these observations and favoring hence the hypothesis of an origin related to snowmelting. In some particular cases, an episode clearly related to snowmelt is perturbed by the contribution of significant rainfall. If the rainfall episode has a duration of minutes to few hours, the regular spectra associated to snowmelt is still recognized (see days 15/5 at Fig 4). In case of very intensive rainfall episodes, the snowmelt signature can not be identified anymore in the seismic data, as shown during the episode starting the 18th June. Note that the same problem would arise when using discharge measurements in a gauge station.

To better discuss the properties of the signal, we have recalculated the spectrograms in segments of 24 hours duration starting at 12:00 UTC, within the frequency band of interest (1.5 and 6.5 Hz). Supporting information S1–S6 Figs show the complete daily spectrograms for the 6 snowmelt seasons investigated so far (1^st^ January– 31th July of each year), allowing to observe the strong differences between each season.

**Fig 5:**
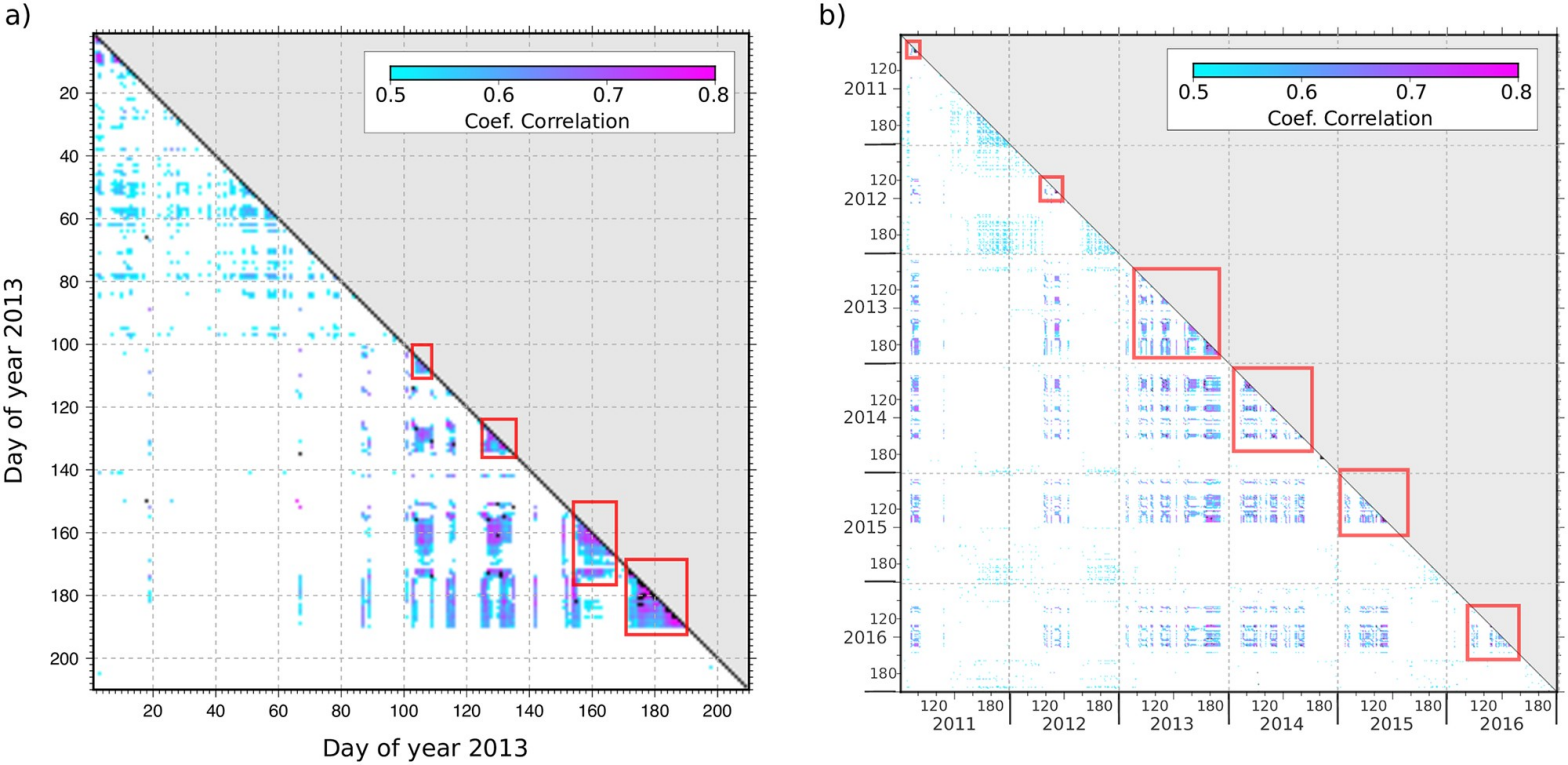
Annual and full dataset spectra correlograms. a) Correlogram of the 24-h spectrograms of the vertical component of the seismic acceleration for days 1–210 of year 2013. Only correlation coefficients exceeding 0.5 are represented. b) Cross-correlogram of the spectrograms for days 80–200 of the 6 years investigated. Red boxes show the activity periods for each season.

Based on the above discussion, we have used the information contained in the daily spectrograms to identify days with active snow melting processes using objective criteria. In a first step, we have explored if the correlation of the spectrograms could be useful to make this identification. To compare the spectrograms we use the correlation routine included in the GMT package [19]. The power spectra values of each spectrogram are arranged as a vector and the correlation between pairs of these vectors is calculated. This procedure allows us to obtain a correlation coefficient for each pair of spectrograms during the investigated period. The final result is a table with the cross-correlation coefficients for each pair of files (days). As an example, Fig 5a shows the correlogram for the first 210 days of year 2013. The spectrograms corresponding to days with river discharges resulting from snowmelt processes are expected to share similar spectral pattern and thus, to have high correlation values. On the contrary, signals related to rain episodes tend to have different duration, initial time and intensity, resulting in low correlation coefficients of the spectrograms. High correlation coefficients (dark colors) are clearly observed for four episodes, corresponding to julian days 102–110, 114–117, 125–135, 154–167 and 170–190. It can be noted that high correlation values are observed not only within each episode, but also between the different identified episodes.

In a second step, the spectrogram cross-correlation analysis is performed jointly for the six available seasons. In order to reduce the computing cost, we consider only the time range including the snowmelt episodes, hence analyzing days 80–200 of each year (21/3–18/7 approx.). The resulting correlogram (Fig 5b) confirms the consistency of the spectral properties across the different years. Annual correlations discussed above are recovered and large interseasonal correlations, appearing as dark shaded colors off of the matrix diagonal, are also identified, hence evidencing that snowmelt related seismic signals have common features across the different seasons.

## Classification of snowmelt episodes

Although the seismic records resulting from discharge increases due to snowmelt share spectral properties, a preliminary inspection of the spectrograms suggests that they can be grouped in different families, each with distinct features. Our hypothesis is that those differences are related to changes in the intensity and pace of the snowmelt processes, which in turn depend on variations in the meteorological and hydrological conditions (temperature, humidity, wind speed, insulation, snow compaction.). Therefore, we consider that gathering this information for long periods of time will provide a useful database for further meteorological and hydrological studies analyzing snowmelt.

In a first step, this classification has been done by visual inspection, just grouping together the daily spectrograms with similar appearance. However, this approach is strongly subjective, as the criteria to define each group, the number of groups to retain and the procedure to relate events and groups for ambiguous cases are difficult to define.

In order to classify the events in a more objective and reproducible way we have decided to use the hierarchical classification tools provided by the Scipy Python library (https://docs.scipy.org/doc/scipy/reference/cluster.hierarchy.html). Hierarchical clustering is a well-established technique that is used in many scientific domains and is often related to unsupervised machine learning procedures. Its objective is to organize a set of input points into a binary tree that groups them accordingly to their similarity. The similarity between the elements, often referred as “distance”, can be estimated using different methods, including the nearest point algorithm, the farthest point algorithm, distances based in average, median, weighted calculations or the method based in the Ward variance minimization algorithm [20]. The procedure starts by computing a similarity matrix for all pairs of input points, considering each of them as a single cluster. At each step, the most similar clusters are combined to form a new cluster that is added to the list, while the original ones are removed and the similarity matrix is then recalculated. Hence, the number of clusters decreases at each step until obtaining a single cluster that encompasses all the input dataset. The final result can be represented by a dendrogram, with all the input values classified in an arborescent schema following their similarity. This graphic can be read as a genealogic or phylogenetic tree, with upper nodes grouping events sharing general properties and lower nodes identifying events with very similar characteristics.

**Fig 6:**
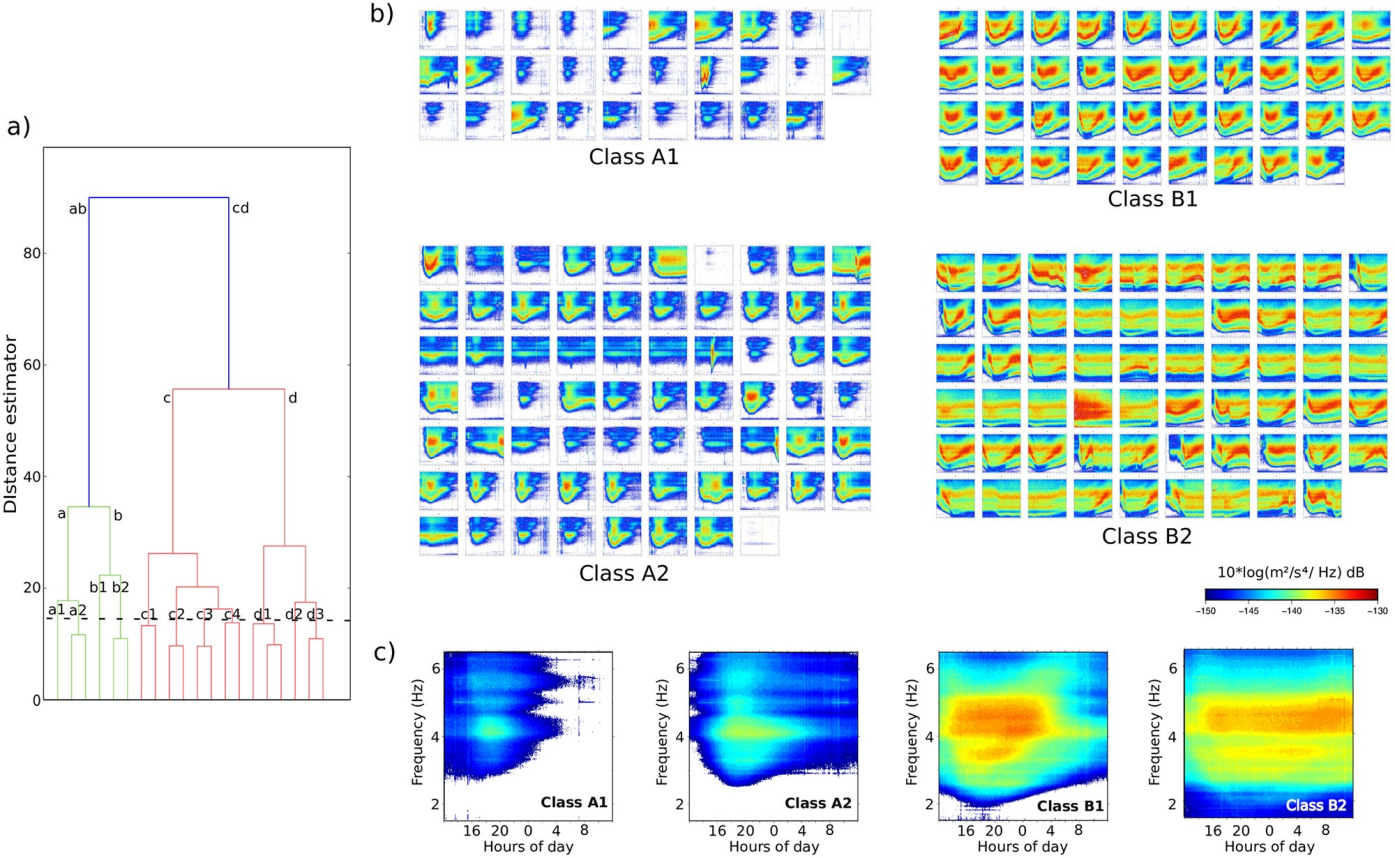
Hierarchical classification of the spectrograms. a) Dendrogram of the hierarchical classification according to the cross-correlation distance. Dashed horizontal black line shows the reference level chosen. Labels refer to each class. b) Daily spectrograms included in each of the four classes related to snowmelt activity. c) Spectrograms built by summation of the individual spectrograms of each class. All the spectrograms share the frequency and time ranges shown in c).

In our case, the input points are the correlation coefficients between pairs of daily spectrograms. The hierarchical clustering will then group those correlation coefficients accordingly to their similarity, measured using the widely used Ward algorithm. The final dendrogram (Fig 6a) allows to identify the different clusters obtained from the classification, hence groping the days with similar snowmelt patterns. The first bifurcation in the dendrogram discriminates two major families with a different amount of individuals. The smaller group (labeled “ab” in Fig 6a) includes 195 days, while the branch labeled “cd” includes 525 days. This bifurcation corresponds to days with or without seismic signal generated by river discharges due to snowmelt. Fig 6b shows all the spectrograms grouped under the “ab” branch, while the rest of the spectrograms are presented at supporting information S7 Fig. Most of the events in the “cd” branch have a low energy level, resulting in whitened spectrograms and making difficult to appreciate the differences among them. However, it can be noted that events in the d2 and d3 clusters have spectrograms with significant energy, corresponding mostly to discharge increases following rainfall events. In the following section, we will focus on analyzing events classed under the “ab” branch. The dendrogram shows that those events can be first classed in two families, labeled “a” and “b”, that in turn are divided in successive subclasses. The number of classes depends on the level value in the dendrogram. As seen in Fig 6a, a value around 30 of the distance estimator will result in a classification with only two classes related to snowmelt, while a level value close to 10 will classify the events in up to 6 different clusters. After several trials, we have selected a level value of 15 of the distance estimator to have a good compromise between homogeneity within each class and differentiation between different classes. Using this reference level, the events related to snowmelt are grouped in four different classes, identified as A1, A2, B1 and B2.

**Fig 7:**
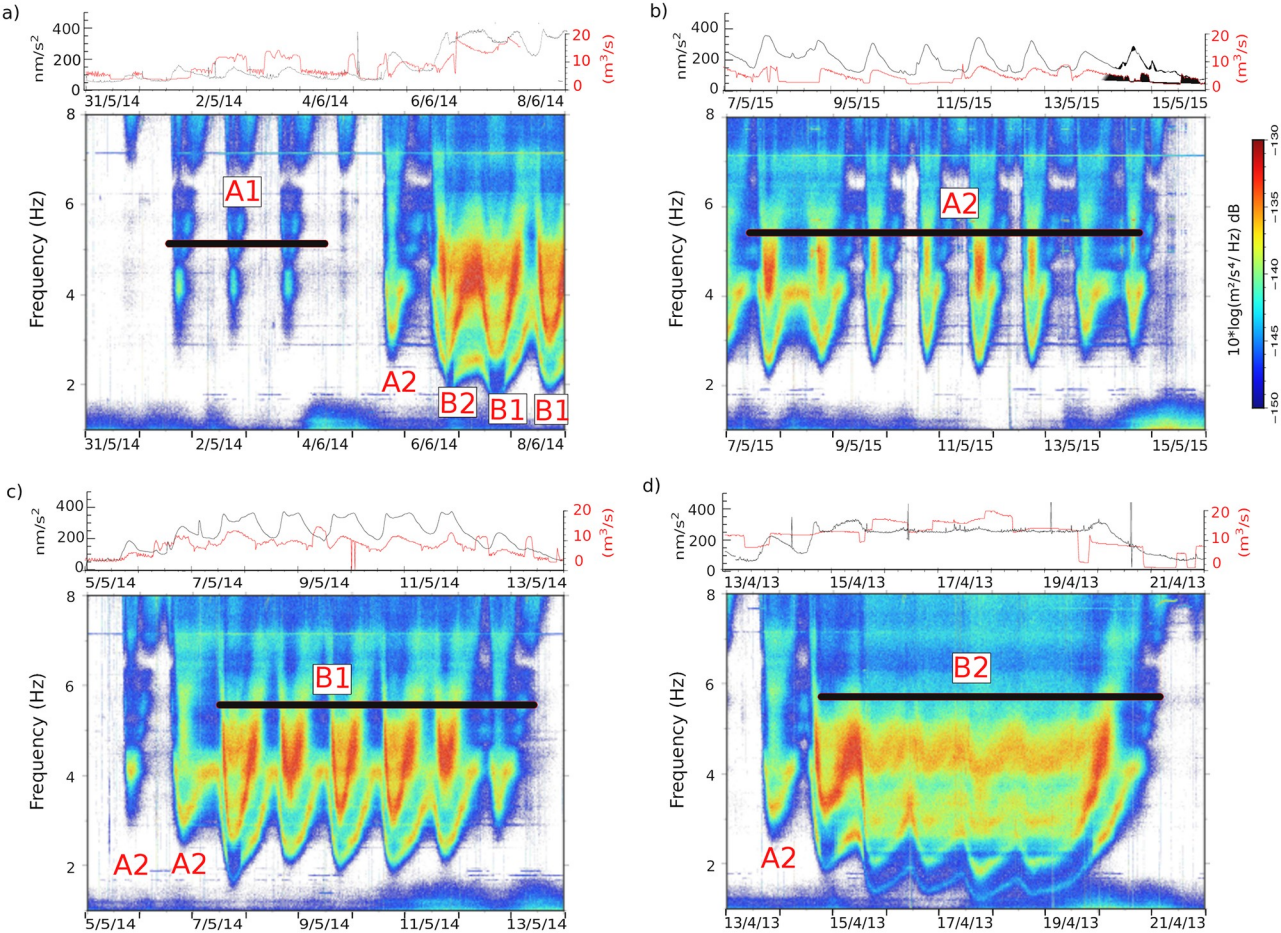
Examples of snowmelt events dominated by one of the described classes. Each example encompasses 9 days. The upper panels show in black the envelope of the seismic signal filtered between 1 and 8 Hz and the discharge values recorded at the hydrological station in red. Lower panels show the corresponding spectrograms between 1 and 8 Hz. Classes attributed to each day are indicated by red labels.

Fig 6c shows the result of adding the spectrograms of each class. Although the summations highlight the differences between classes, they produce a significant smoothing of the time-frequency variations of the spectrograms. To better discuss the properties of the different classes, Fig 7 shows examples of snowmelt episodes dominated by events of each of the four classes. The upper panels on the figure compare the seismic data with the available discharge data recorded by the A271 CHE gauge station. It should be noted that the discharge time series have a significant number of inconsistencies, including periods without data and spikes, while seismic data provide a more continuous recording. It is also worthy to note that hydrological data is only able to recover partially the 24 hours cycle expected from snowmelt, clearly evidenced by the seismic spectra. This can be explained by the effect of the small pond located between the seismic and the hydrological stations. Although this pond is not devoted to water flow management, it can mask the tiny discharge changes related to low intensity snow melting. Therefore, for this particular location, seismic data provides a more accurate description of the snowmelt process.

For events in classes A1 and A2 the seismic energy increases sharply around 14:00 GMT, remains at relatively high levels for about 6–16 hours, then vanishes smoothly to finally increase again. The signals recorded during each cycle are hence clearly separated by periods without relevant seismic energy. A1 events show significant energy for frequencies above 3 Hz, with a peak between 16:00 and 23:00 at frequencies near 4 Hz. The daily spectrograms for A2 class events show a well-defined V-shaped pattern, starting with a dominant frequency around 4 Hz close to 14:00, shifting down quickly to reach minimum values between 2.5 and 3 Hz and increasing smoothly later on to reach its original value. In most of the cases a large part of the total energy appears between 4 and 5 Hz during the afternoon and night hours (16:00–22:00). Comparing our data with the discharge measured at the hydrological station, the events of class A1 correspond to mean discharge of around 4 m^3^/s with maximum values lower than 10 m^3^/s, while those of class A2 correspond to mean discharge values of around 8 m^3^/s with maximum values slightly higher than 10 m^3^/s (Fig 7a and 7b).

Classes B1 and B2 are characterized by higher and sustained levels of seismic signal during the whole day. Events in the B1 class have a V-shaped variation similar to those at class A2, but showing higher energy levels and covering a lower frequency range. The cycle starts between noon and 14:00 with a dominant frequency around 3.5 Hz, reaches a minimum close to 2 Hz 6–8 hours later and increases again to 4 Hz in the early morning. As in the previous case, a large part of the energy appears in a frequency band ranging between 4 and 5 Hz during the central part of the daily cycle. Most of the events in the B2 class do not show a clear V-shape time variation, although this pattern is still visible for the lowermost energetic frequencies, near 1.8 Hz. Most of the energy is located in this case within two more or less continuous bands between 3–3.5 Hz and 4.25–5 Hz respectively. Events classed as B1 typically correspond with discharge values between 10 m^3^/s and 15 m^3^/s, while B2 events correspond to sustained values of discharge exceeding 15 m^3^/s (Fig 7c and 7d).

**Fig 8:**
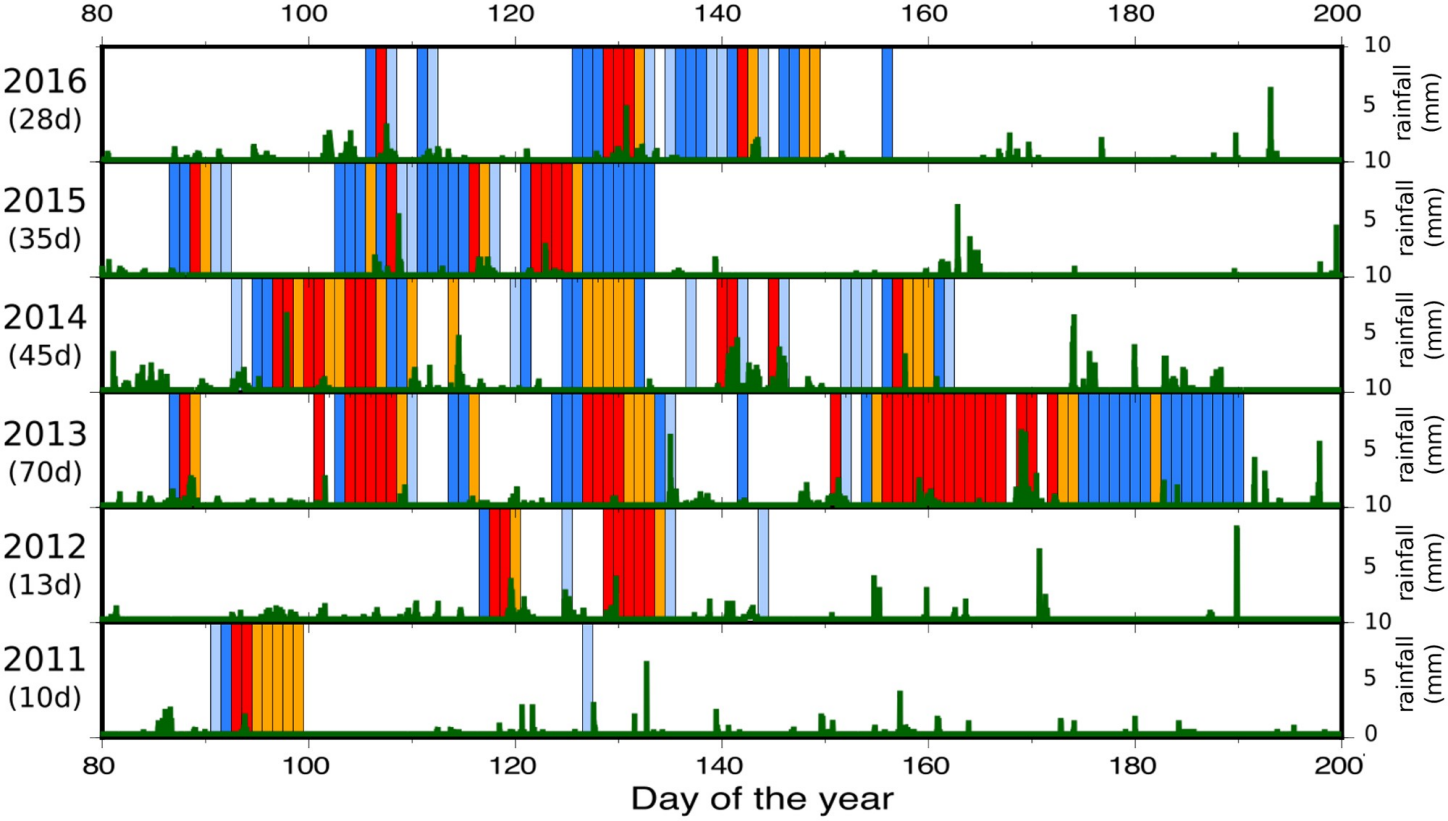
Active snowmelt periods for each investigated year. Light and dark blue bars are for A1 and A2 events, while orange and red bars show B1 and B2 ones. Green bars are for 10 or 15 minutes rainfall measurements at the A271 gauge station.

The results of the identification and classification of the snowmelt related episodes for the 6 years under study are compared with the rainfall measurements at the gauge station A271 in Fig 8. It is difficult to evaluate the number of false positive and negative of our procedure, as the snowmelt activity can only be deduced indirectly. However, we have compared the results of the hierarchical classification presented here with a visual inspection of the spectrograms and the meteorological and hydrological data, concluding that a maximum of 2 or 3 days per year are probably misidentified, representing less that the 5–10% of the typical number of annual identifications. For a slightly higher number of events, coetaneous rainfall and snowmelt activity results in over classing of the event. In order to document this feature, S2 to S7 Figs show the results of our classification overprinting the daily spectrograms.

The number of days with snowmelt activity is highly variable, ranging between 10 days during the driest season (2011) and 65 days for the 2013 season. Years 2014–16 suggest that a value around 35 days distributed in 2–3 major episodes is representative of this location. Typically, the snowmelt is concentrated between April and May, although during 2013 the seismic signature associated with snowmelt reaches the beginning of July. The 2011 season, drier than average, shows a single snowmelt episode in early April lasting 9 days (1^st^– 9^th^ April). In 2012, again a dry season, two events lasting 4 and 7 days can be identified between the 25^th^ April and the 16^th^ May. The 2013 season has a clearly different character, with up to 5 episodes of snowmelting identified in the seismic data, extending from late March to early July. The last episode (31^th^ May– 9^th^ July) has an unusual duration exceeding 35 days, with just three days without evidence of snowmelting. The 2014 season shows a rather different snowmelt evolution, with the longer episode observed between the 5^th^ and the 20^th^ April, followed by a second episode occurring between 5^th^ and 13^th^ May. By late May, a couple of events are interpreted as the joint result from snowmelt and rainfall. The last episode of melting for this season is identified at early June (1^st^– 12^th^). The 2015 season has the largest estimation of accumulated snow for the full Aragón River basin according to the available hydrological models (http://www.chebro.es/contenido.visualizar.do?idContenido=2233&idMenu=2190). The first snowmelt episode appears in late March, slightly earlier than usual. Most of the snowmelt concentrates in two episodes separated by 3–4 days between mid April and mid May. Quite surprisingly, the snowmelt ends by mid May, as for the case of the dry 2011 and 2012 seasons. During 2016 most of the snowmelt concentrates in a long episode spanning from 4^th^ May to 28^th^ May. Fig 8 shows also that the discharge measured at the CHE A271 gauge station conforms closely to the seismic data, with larger discharges corresponding to B1 and B2 seismic events.

## Discussion

For snowmelt episodes, the 24-hours periodicity in the discharge of the draining river is explained by the dynamics of snow melting in Alpine regions, where snowmelting water is not typically drained on surface but percolates through the snowpack [18]. Therefore, a time delay exists between the hours with active melting and the discharge increase in the river. The snowmelt process is only active during day-time and hence the large discharge is expected during the central hours of the day, with a gradual decrease in the melt stream during the night. The seismic data suggest that for the class A1 and A2 events the discharge stops almost completely during late night and morning hours, while for those classified as B1 and B2 there is a certain continuity during the whole 24 hours cycle. Typical snowmelt hydrographs described in the literature show discharge increasing sharply to reach maximum values between 15:00 and 21:00 local time and then gradually decrease. The seismic signal of the events classed as A1, A2 and B1 match closely these characteristics, with the daily cycle starting at 14:00, showing a minimum value on the amplitude and the dominant frequency around 18:00 and increasing again till the morning. For events classed as B2 the daily periodicity is still visible, although the energy variations are more subtle. We attribute the differences between snowmelt events in the different classes to the size and timing of the associated river discharge and, consequently, to the intensity of the melting process. Note that these variations are difficult to infer from the available gauge station data, as this station is located downstream of a small regulatory pond which can mask the tiny discharge variations associated with snowmelt. Additionally, gauge stations based on water level measurements are exposed to changes in the river channel geometry that can perturb the discharge value estimation and explain the inconsistencies observed in the data (Fig 7).

Most authors relate the origin of the seismic signal observed in fluvial seismology to water turbulence induced by the stream, bed load particles impacting the river bed or the joint effect of the two processes [21]. Although relevant theoretical models have been presented recently [22–24], the relative importance of bedload and water turbulence as sources of the seismic signals remains a matter of debate. Our observations provide some additional observations to this discussion.

The dominant frequency during snowmelt events involving low discharges (classes A1, A2 and B1) reach its minimum value when the seismic amplitude and the discharge are maximal. On the contrary, for the snowmelt events involving larger discharges (class B2) the correlation between frequency and amplitude is less clear, but the available data suggest that minimal frequencies tend to correlate with relatively lower amplitudes (Fig 7d). Experiments in controlled sites [25] have shown that when large particles are mobilized the dominant frequency in the seismic data decreases. Following this observation, we can interpret that during snowmelt events involving moderate discharges, the recorded signal is related to the size of the mobilized bed load particles; large bedload particles are only mobilized during the periods of maximal discharge, hence resulting in large seismic amplitude and lower dominant frequencies. Consistently, the smooth frequency increase observed during the coda of all the hydrological events analyzed in this contribution can be related to the progressive decrease in discharge and thus in the size of the mobilized particles.

It must be pointed that overtones are clearly observed in the spectrograms during snowmelt episodes. This kind of overtones has been observed in glaciers moulins feeding subglacial drainage systems [26], but are not usually observed in river seismology. We suggest that the presence of these overtones is related to the specific characteristics of the Aragón River channel in the vicinity of the recording site (Fig 1). Regular water height variations in open water channels can generate similar resonance waves, in particular for deep and short channels with smooth walls and relative low flows [27]. The Aragón River channel near the recording site is narrow and entrenched into the basement rocks (Fig 1), hence satisfying those conditions. Nevertheless, an accurate modeling, out of the scope of this contribution, would be needed to confirm this hypothesis.

## Conclusions

Seismic data recorded in the Canfranc Underground Laboratory allow monitoring the time occurrence and properties of the snowmelt stages for each hydrological season. A hierarchical classification of the seismic spectra is used to identify the existence of different snowmelt styles, related in turn to different meteorological scenarios, and to quantify its occurrence using objective criteria. Thus, the seismic records provide a valuable tool to study the long term variations of the snowmelt processes in high altitude mountain settings and can be considered as a proxy for the meteorological factors affecting snowmelt.

The variations in the dominant frequency during snowmelt episodes observed in the spectrograms are related to the relative contribution of water turbulence and sediment transport as sources of the seismic signals. Our results suggest that during relatively large and sustained floods, the dominant source of seismic signal is water turbulence, while for lower discharges the frequency variations are due to the different size of the bedload particles mobilized during the different phases of the discharge. However, further theoretical work is needed to fully explain the observations.

Snowmelt in the Aragon River valley can typically be observed during 30–40 days distributed in 2–3 main episodes between March and June. Significant differences appear between the six snow seasons analyzed in this study, including examples of dry seasons (2011 and 2012) and of seasons with large snowmelt accumulation (2013), with snowmelting still active in early July.

The classification of the daily spectrograms using an hierarchical algorithm has proved to be useful to identify different clusters of snowmelt related events, associated with distinct snowmelting styles. This has allowed us a first investigation of the time evolution of the snowmelt process during each season, which has a clear potential for future meteorological and hydrological studies.

This work shows that continuous seismic recording is suitable for long-term hydrological studies, since it provides detailed information of daily and seasonal cycles. Comparing with typical hydrological gauge stations, seismic instruments have the advantage of not being exposed to changes in the river channel geometry. Seismometers can be installed upstream of regulation ponds, hence avoiding their effect in hydrological stations. As the installation of multiple seismic stations in a same region during a complete snowmelt season is feasible, a detailed study of the characteristics of snowmelt in different sections of a basin can be envisaged. However, as the frequency range of the seismic signals generated by the river discharge is within the band affected by anthropogenic activities, seismic monitoring of snowmelt related activity requires sites far from areas with significant human activity.

## Supporting information

S1 Fig Daily spectrograms for year 2011 (Julian days 55–200). Each spectrogram begins at 12:00 UTC and end at the same hour the next day. Frequency range: 1.5–6.5 Hz.
(PDF)

S2 Fig Daily spectrograms for year 2012 (Julian days 1–200). Each spectrogram begins at 12:00 UTC and end at the same hour the next day. Frequency range: 1.5–6.5 Hz.
(PDF)

S3 Fig Daily spectrograms for year 2013 (Julian days 1–200). Each spectrogram begins at 12:00 UTC and end at the same hour the next day. Frequency range: 1.5–6.5 Hz.
(PDF)

S4 Fig Daily spectrograms for year 2014 (Julian days 1–200). Each spectrogram begins at 12:00 UTC and end at the same hour the next day. Frequency range: 1.5–6.5 Hz.
(PDF)

S5 Fig Daily spectrograms for year 2015 (Julian days 1–200). Each spectrogram begins at 12:00 UTC and end at the same hour the next day. Frequency range: 1.5–6.5 Hz.
(PDF)

S6 Fig Daily spectrograms for year 2016 (Julian days 1–200). Each spectrogram begins at 12:00 UTC and end at the same hour the next day. Frequency range: 1.5–6.5 Hz.
(PDF)

S7 Fig Daily spectrograms for classes C and D, not showing evidences of snowmelt activity. (PDF)
