# Supplementary material for: Hierarchical classification of snowmelt episodes in the Pyrenees using seismic data
Source: PLoS One. 2019 Oct 10;14(10):e0223644. doi: 10.1371/journal.pone.0223644 (PMC6786603; doi:10.1371/journal.pone.0223644)
Supplement: S4 Fig — Each spectrogram begins at 12:00 UTC and end at the same hour the next day. Frequency range: 1.5–6.5 Hz. (PDF) [file pone.0223644.s004.pdf]

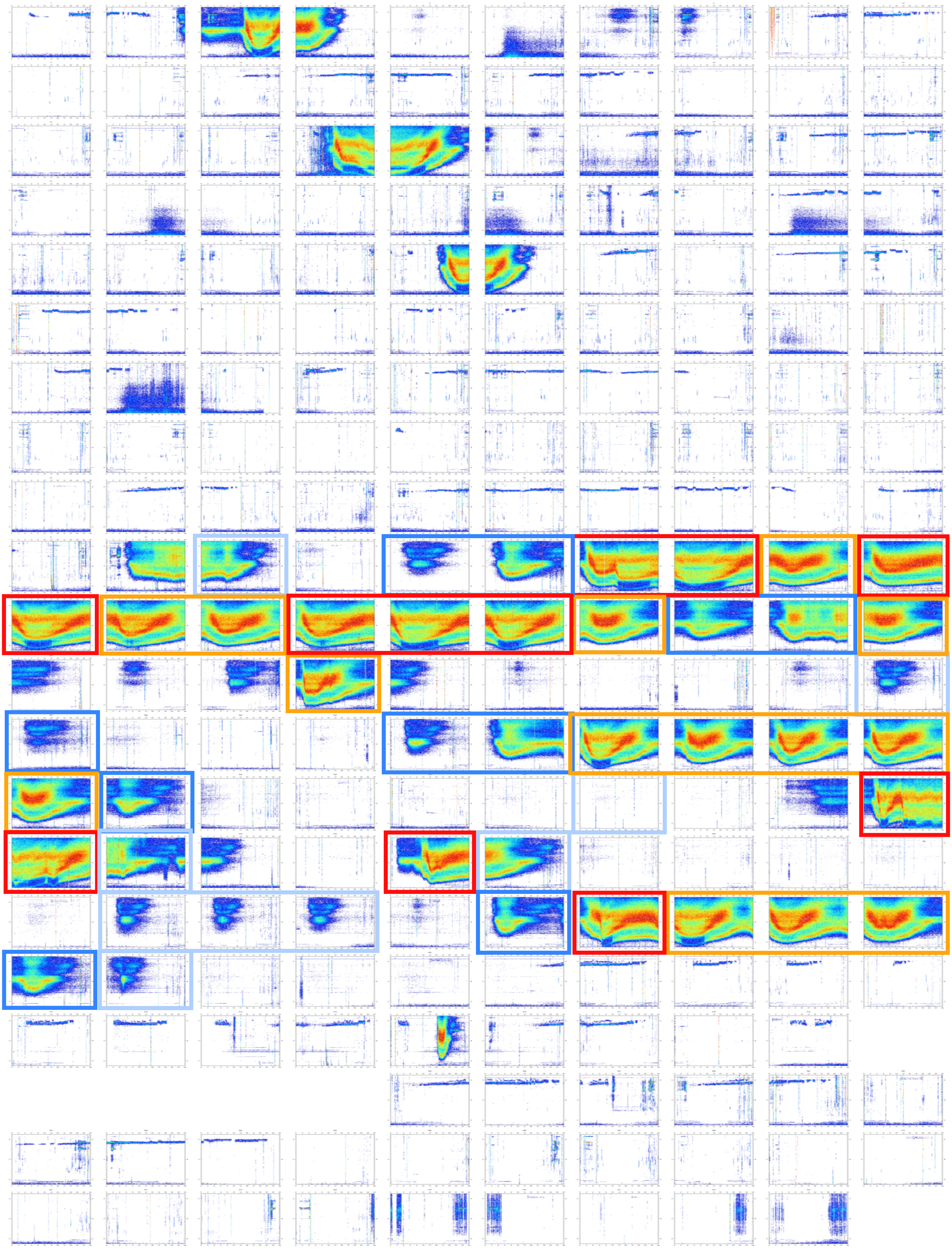

Supplemental Figure S4: Daily spectrograms for year 2014 (Julian days 1-200). Each spectrogram begins at 12:00 UTC and end at the same hour the next day. Frequency range: 1.5 - 6.5 Hz. Color boxes show the results from the hierarchical classification (see Fig 8).
