# Supplementary material for: Hierarchical classification of snowmelt episodes in the Pyrenees using seismic data
Source: PLoS One. 2019 Oct 10;14(10):e0223644. doi: 10.1371/journal.pone.0223644 (PMC6786603; doi:10.1371/journal.pone.0223644)
Supplement: S7 Fig — (PDF) [file pone.0223644.s007.pdf]

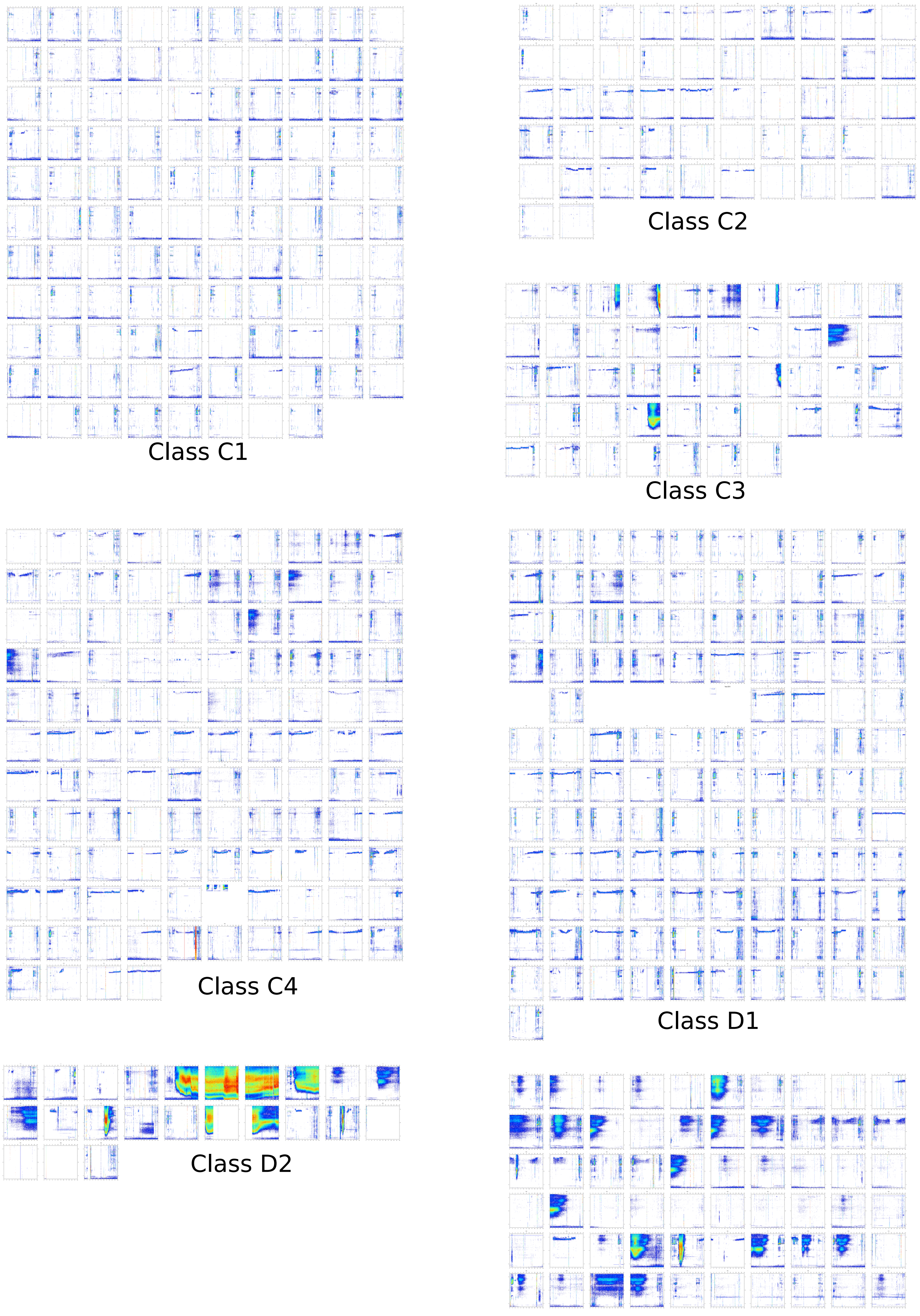

Supplemental Figure S7: Daily spectrograms for classes C and D, not showing evidences of snowmelt activity.
